# The Raman-Derived Carbonization Continuum: A Tool to Select the Best Preserved Molecular Structures in Archean Kerogens

**DOI:** 10.1089/ast.2015.1392

**Published:** 2016-06-01

**Authors:** Frédéric Delarue, Jean-Noël Rouzaud, Sylvie Derenne, Mathilde Bourbin, Frances Westall, Barbara Kremer, Kenichiro Sugitani, Damien Deldicque, François Robert

**Affiliations:** ^1^ IMPMC Sorbonne Universités—MNHN, UPMC Univ Paris 06, UMR CNRS 7590, IRD UMR 206, Paris, France.; ^2^ Laboratoire de Géologie de l'ENS, UMR CNRS 8538, Paris, France.; ^3^ Sorbonne Universités, UPMC Univ Paris 06, CNRS, UMR 7619 METIS, CC 105, Paris, France.; ^4^ Centre de Biophysique Moléculaire, UPR CNRS 4301, Orléans, France.; ^5^ Institute of Paleobiology, Polish Academy of Sciences, Warszawa, Poland.; ^6^ Department of Environmental Engineering and Architecture, Graduate School of Environmental Studies, Nagoya University, Nagoya, Japan.

## Abstract

The search for indisputable traces of life in Archean cherts is of prime importance. However, their great age and metamorphic history pose constraints on the study of molecular biomarkers. We propose a quantitative criterion to document the thermal maturity of organic matter in rocks in general, and Archean rocks in particular. This is definitively required to select the best candidates for seeking non-altered sample remnants of life. Analysis of chemical (Raman spectroscopy, ^13^C NMR, elemental analysis) and structural (HRTEM) features of Archean and non-Archean carbonaceous matter (CM) that was submitted to metamorphic grades lower than, or equal to, that of greenschist facies showed that these features had all undergone carbonization but not graphitization. Raman-derived quantitative parameters from the present study and from literature spectra, namely, R1 ratio and FWHM-D1, were used to draw a carbonization continuum diagram showing two carbonization stages. While non-Archean samples can be seen to dominate the first stage, the second stage mostly consists of the Archean samples. In this diagram, some Archean samples fall at the boundary with non-Archean samples, which thus demonstrates a low degree of carbonization when compared to most Archean CM. As a result, these samples constitute candidates that may contain preserved molecular signatures of Archean CM. Therefore, with regard to the search for the oldest molecular traces of life on Earth, we propose the use of this carbonization continuum diagram to select the Archean CM samples. Key Words: Archean—Early life—Kerogen—Raman spectroscopy—Carbonization. Astrobiology 16, 407–417.

## 1. Introduction

The biological origin of some Archean carbonaceous matter (CM) is still debated (Schopf and Packer, 1987; Brasier *et al.*, 2002; Lindsay *et al.*, 2005; Marshall *et al.*, 2012). Indeed, although most non-Archean CM is usually considered to be of biological origin, abiotic processes such as Fischer-Tropsch-type synthesis and siderite decomposition are often thought to account for Archean CM formations (van Zuilen *et al.*, 2002, 2003; McCollom and Seewald, 2006; see van Zuilen *et al.*, 2007, and references therein for more details about abiotic formation of CM). Molecular characterization of Archean CM has recently been recognized as a promising tool with which to discriminate between biotic and abiotic CM (Brocks *et al.*, 2003; Marshall *et al.*, 2007; Derenne *et al.*, 2008). Unfortunately, in most samples, multiple sources of postdepositional CM and the extensive impact of metamorphism have resulted in the masking or elimination of molecular structures and a lack of univocal molecular biosignatures (Bourbin *et al.*, 2012a; French *et al.*, 2015). Quantifying the degree of alteration of Archean CM was thus of prime interest in the search for molecular biosignatures in the oldest cherts on Earth (Marshall *et al.*, 2012). Indeed, as stressed by French *et al.* (2015), “future exploration for Archean biomarkers should screen for rocks with milder thermal histories.”

In this respect, the thermal alteration of CM is known to be driven by two reactions, namely, carbonization and graphitization (Oberlin, 1984, 1989). Carbonization is characterized by (i) chemical changes that consist of the relative enrichment of CM in aromatic structures (aromatization) due to the loss of oxygenated groups and other chemical groups containing heteroelements and aliphatic units, and (ii) structural changes resulting in the formation of nanometer-sized polyaromatic layers that tend to stack into structural units (Oberlin, 1984, 1989; Rouzaud *et al.*, 2012, 2015). In natural environments, carbonization takes place in the 100–500°C temperature range (Mrozowski, 1988a, 1988b; Lahfid *et al.*, 2010). Graphitization is a process whereby the aromatic skeleton is reorganized, yielding hexagonal graphite with triperiodic order and subsequent crystalline growth (Oberlin, 1984; Lahfid *et al.*, 2010; Rouzaud *et al.*, 2012, 2015; Charon *et al.*, 2014). In natural carbons, graphitization takes place at higher temperature than carbonization and only in the presence of pressure (Oberlin 1984, 1989).

In recent years, Raman spectroscopy has become a favored technique with which to investigate CM evolution by way of carbonization and graphitization processes (Lahfid *et al.*, 2010; Rouzaud *et al.*, 2012, 2015; Charon *et al.*, 2014). Raman spectra of CM exhibit two broad bands that are respectively assigned to defects (D) and graphite (G). Upon carbonization, these bands narrow, and their intensity ratios I(D)/I(G) increase, whereas I(D)/I(G) decrease during graphitization (Bernard *et al.*, 2010; Rouzaud *et al.*, 2012, 2015; Charon *et al.*, 2014). Because carbon maturation is an irreversible process, CM records its highest thermal maturity stage (Beyssac *et al.*, 2002).

In the present study, we investigated through Raman spectroscopy the chemical and structural features of kerogens isolated from cherts that underwent no, or low, metamorphism that ranges from prehnite-pumpellyite to greenschist facies and with ages that range from 0.05 to 3.5 Ga. The Raman spectroscopy data will be discussed in light of results from elemental analysis, solid-state ^13^C nuclear magnetic resonance (NMR), and high-resolution transmission electron microscopy (HRTEM). Utilizing data derived from Raman spectra in the literature, we propose a framework with which to select the most favorable samples in the search for molecular traces of life.

## 2. Material and Methods

### 2.1. Samples

Seventeen cherts of various ages, metamorphic facies, and geographical origins were studied. Cherts were selected because of their low porosity, which makes their organic matter less prone to postdeposition contamination. The sample characteristics are given in Table 1. All cherts underwent no, or low, metamorphism ranging from prehnite-pumpellyite to greenschist facies and with ages ranging from 0.05 to 3.5 Ga (Table 1).

**Table 1.** Characteristics of the Cherts Studied (Identified by Numbers 1 to 17)

*N°*	*Chert reference*	*Age (Ga)*	*Geological unit, locality*	*Estimated metamorphic grade*	*Described in*
1	Clarno (PPRG456)^a^	0.05	Clarno Formation, John Day Basin Tectonic Unit, Oregon, USA	n.m.	Walter *et al.*, 1983
2	Rhynie (1 of 9/13/83)^b^	0.4	Rhynie, Dryden Flags Formation, Grampian Highlands, Aberdeenshire, Scotland	n.m.	/
3	Zalesie Nowe^c^	0.42	Zalesie Nowe, Holy Cross Mountains, Bardo Syncline, Poland	p.p. to p.a.	Kremer and Kazmierczak, 2005
4	Żdanow^c^	0.42	Żdanow, Bardzkie Mountains, Sudetes Mountains, Poland	p.p. to p.a.	Kremer, 2006
5	Döbra^c^	0.42	Döbra, Franconian Forest, Bavaria, Germany	lower g.s.	Kremer *et al.*, 2012
6	Gunflint (3 of 06/30/84)^b^	1.9	Gunflint Iron Formation, Port Arthur Homocline Tectonic Unit, Ontario, Canada	lower g.s.	Awramik and Barghoorn, 1977; Marin-Carbonne *et al.*, 2012
7	Gunflint (PPRG134)^b^	1.9	Gunflint Iron Formation, Port Arthur Homocline Tectonic Unit, Ontario, Canada	lower g.s.	/
8	Gunflint (1 of 08/23/86)^b^	1.9	Gunflint Iron Formation, Port Arthur Homocline Tectonic Unit, Ontario, Canada	lower g.s.	Beaumont and Robert, 1999
9	Rietgat (SB023)^b^	2.65	Rietgat Formation, Platberg Group, Ventersdorp Supergroup, South Africa	lower g.s.	/
10	Farrel Quartzite (GGR2)^d^	3.0	Mount Goldsworthy–Mount Grant area, Pilbara Craton, Australia	lower to mid g.s.	/
11	Farrel Quartzite (GRW10)^d^	3.0	Mount Goldsworthy–Mount Grant area, Pilbara Craton, Australia	lower to mid g.s.	/
12	Farrel Quartzite (ORW9)^d^	3.0	Mount Goldsworthy–Mount Grant area, Pilbara Craton, Australia	lower to mid g.s.	/
13	Farrel Quartzite (GFWEX 1-1b)^d^	3.0	Mount Goldsworthy–Mount Grant area, Pilbara Craton, Australia	lower to mid g.s.	/
14	Farrel Quartzite (MGTKS1 up)^d^	3.0	Mount Goldsworthy–Mount Grant area, Pilbara Craton, Australia	lower to mid g.s.	/
15	Josefsdal (99SA07)^e^	3.3	Josefsdal Valley, Kromberg Formation, Onverwacht Group, Barberton greenstone belt, South Africa	g.s.	Westall *et al.*, 2006
16	Middle Marker (07SA22)^e^	3.4	Middle Marker, Komati Formation, Barberton greenstone belt, South Africa	g.s.	/
17	Dresser (PPRG006)^a^	3.5	Dresser Formation (former Towers Formation), Warrawoona Group, Pilbara Block, Australia	p.p. to lower g.s.	Walter *et al.*, 1983; Derenne *et al.*, 2008

The cherts were collected by ^a^J.W. Schopf, ^b^S.M. Awramik, ^c^B. Kremer, ^d^K. Sugitani, and ^e^F. Westall. The metamorphic facies are indicated as follows: n.m. = non-metamorphosed; p.p. = prehnite-pumpellyite; p.a. = pumpellyite-actinolite; g.s. = greenschist.

The Clarno Formation (*ca.* 0.05 Ga) is situated in the John Day Basin in northern Oregon, United States of America. It consists of thick layers of various rocks that range from volcanic to sedimentary and formed within an extensional basin or a series of basins near a volcanic arc complex. We examined a black chert sample (n°1; Table 1) that formed in a marsh environment in close proximity to hot springs. The hot springs provided silica, which precipitated to form bedded cherts (Arnold and Daugherty, 1964). Clarno black chert contains millimeter- to centimeter-scale permineralized fossils of organic materials such as wood tissue, fungi remnants, and diffused organic matter.

The Rhynie sample (n°2; Table 1) was collected in the Dryden Flags Formation, Aberdeenshire, North-East Scotland. This Devonian sample is characterized by a microcrystalline silica matrix that formed by way of a subaerial hot spring system (Rice *et al.*, 1995). Formation of silica sinters favored the exceptional preservation remnants of continental life, and the studied sample contains a wide variety of CM from fungi, algae, spores, and woody remnants.

Zalesie Nowe, Żdanów (Poland), and Döbra cherts (Germany; n°3–5; Table 1) are early Silurian samples that are representative of typical Paleozoic primary cherts composed of cryptocrystalline and mostly homogeneous quartz with a small admixture of phyllosilicate minerals. The cherts are distinctly laminated and consist of well-defined, horizontally extended undulating laminae that are 10–40 μm thick and composed of amorphous dark brown to brownish-red organic material. Organic matter has been identified mostly as fossil remnants of algae and benthic cyanobacterial mats. Graptolites indicate a Llandovery (early Silurian) age for all samples.

The Gunflint cherts (n°6–8; Table 1) were collected in the Gunflint Formation, Port Arthur homocline, Ontario, Canada. This 1.9 billion-year-old formation is composed of alternation between banded iron formation rocks and silica cherts. The three studied black cherts are dominated by cryptocrystalline quartz. They also comprise spheric and filamentous structures.

The Rietgat chert (n°9; Table 1) was sampled in the Ventersdorp Conglomerate Supergroup, Platberg Group (South Africa), which comprises a succession of volcanic and sedimentary rocks. The Rietgat sample (*ca.* 2.6 Ga) represents fluvial and/or lacustrine silicified sediments in which the occurrence of algal matter was suggested (Buck, 1980).

Farrel Quartzite samples (n°10–14; 3.0 Ga; Table 1) were collected from the Goldsworthy greenstone belt in the Pilbara Craton, Western Australia. Two samples (GFWEX1-1b, MGTKS1 up) are bedded black chert and contain microfossils (Sugitani *et al.*, 2007, 2009; House *et al.*, 2013, and references therein). They are assumed to have deposited in a shallow evaporitic basin with input of hydrothermal fluids (Sugahara *et al.*, 2010). GGR2 is a black chert interbedded with sandstone from the lower unit of the Farrel Quartzite. ORW9 and GRW10 are laminated black cherts from the cherty succession, which conformably overlies the Farrel Quartzite that is assigned to the Cleaverville Formation.

The Josefsdal chert (n°15; 3.3 Ga; Table 1) was sampled from a chert horizon situated in the Barberton greenstone belt, Onverwacht Group, located in the upper part of the Josefsdal Valley, South Africa. The Josefsdal chert sample consists of silicified volcaniclastic sediments. It is laminated and contains phyllosilicate grains and silica veins (Westall *et al.*, 2006).

The Middle Marker chert (n°16; 3.4 Ga; Table 1) was sampled in the Barberton greenstone belt, South Africa. It consists of silicified detrital sediment comprising volcanic grain, fluid inclusions, and CM floccules (Bourbin *et al.*, 2012b).

The Dresser chert (n°17; 3.5 Ga; Table 1) was collected in the Towers Formation at North Pole B Deposit Mine, Warrawoona Group, Pilbara Craton, Australia (Walter *et al.*, 1983). This sample is a secondary chert that formed by the accumulation of carbonate sediments that were then silicified by hydrothermal fluids. This chert is characterized by microcrystalline silica with dispersed CM.

### 2.2. Methods

The kerogen was isolated from the cherts by using the classical HF/HCl protocol (Durand and Nicaise, 1980). The samples were crushed in a mechanical crusher, which was previously cleaned with ethanol. The soluble compounds were first extracted by using a dichloromethane/methanol (2/1: v/v) solvent mixture. The remaining powder was then submitted to a first acidic treatment with HCl 6 *N* and then to a second acidic treatment with a HF (40%)/HCl (6 *N;* 2/1: v/v) mixture. A final acidic treatment was then conducted with HCl 6 *N* at 60°C to dissolve any fluorides that may have been formed during the previous acidic treatment.

Ash content and elemental analyses for carbon (±0.4%) and hydrogen (±0.2%) contents were conducted by the SGS Company using calcination at 1000°C and thermal conductibility, respectively.

Cross-polarization/magic angle spinning (CP/MAS) solid-state ^13^C NMR spectroscopy was run on a Bruker Avance 400 spectrometer using a 14 kHz spinning rate to spin out chemical anisotropy and avoid spectrum disturbance by spinning side bands. Recycle and contact times were 10 s and 1 ms, respectively.

**FIG. 1.**
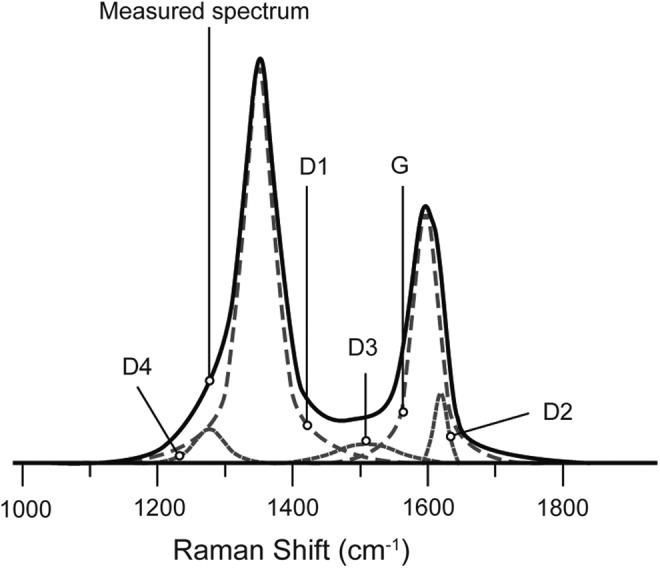
Decomposition of the Raman spectrum of CM.

Raman spectra were obtained with a Renishaw InVIA microspectrometer, equipped with a 514.5 nm Spectra Physics argon laser at 20 mW. The laser was focused on the sample by using a DMLM Leica microscope with a 100× objective. The laser power at the sample surface was set at below 1 mW to prevent thermal alteration. The signal was detected by a Peltier cooled RENCAM CCD detector. The spectrometer was calibrated with a silicon standard before each session. As proposed by Sadezky *et al.* (2005), Raman spectra were decomposed into a combination of five Lorentzian/Gaussian bands (namely, D1, D2, D3, D4, and G; Fig. 1). In this decomposition, the height of the D1 band (occurring at *ca.* 1350 cm^−1^) was fixed to be that of D, and the heights of the D2 to D5 bands were adjusted to obtain the best fit. Various Raman parameters can be determined: the full width at half maximum of the D1 (FWHM-D1) and of the G (FWHM-G) bands, and the intensity (band height) ratio of the defect (D1) and the graphite (G) bands, termed the R1 ratio (Beyssac *et al.*, 2002).

As the heights of D and D1 are usually identical, R1 ratios could be directly determined graphically from literature Raman spectra and then compared to our data. FWHM-D1 values were also graphically estimated when values were lacking in the literature. The graphical procedure involves two biases in the determination of these parameters. First, it may enhance error in the estimation of the FWHM-D1 as D3, D4, and D5 bands are not considered. Second, it may underestimate the R1 ratio when a D2 is well defined. To assess the reliability of R1 and FWHM-D1 graphical determination, we compared the values obtained by both graphical determination and spectra decomposition. Results obtained from the two procedures were highly correlated for both R1 (*R*^2^ = 0.96) and FWHM-D1 (*R*^2^ = 0.94), validating the graphical determination of these parameters. HRTEM observations were carried out with a Jeol 2011 microscope operating at 200 keV. An image analysis technique that was initially developed for HRTEM images of disordered industrial carbons was then applied (Rouzaud and Clinard, 2002).

## 3. Assessment of the Thermal Maturity

**FIG. 2.**
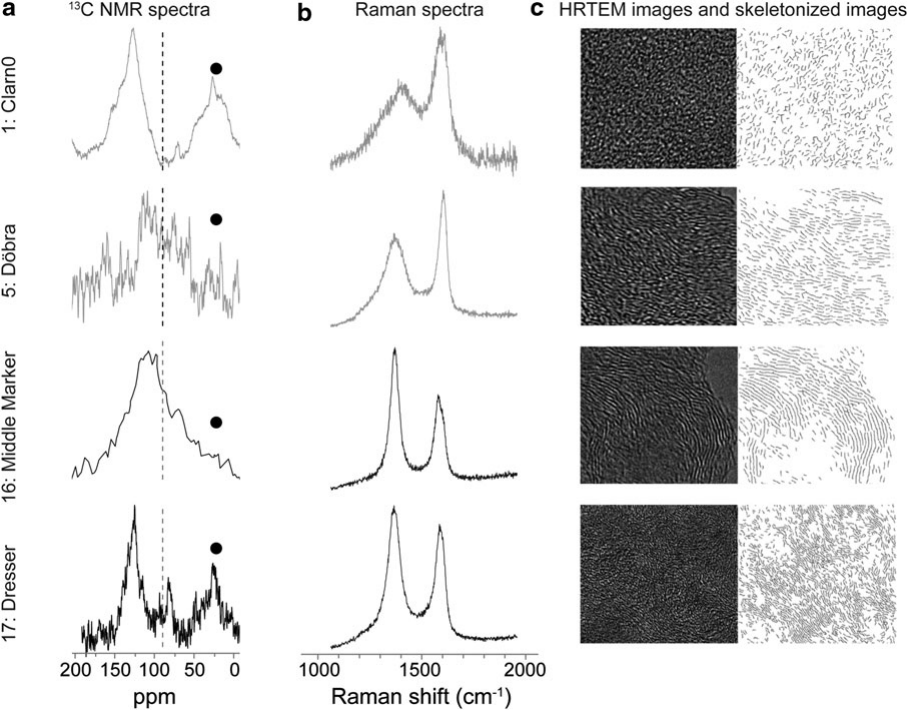
(**a**) ^13^C NMR spectra, (**b**) Raman spectra, (**c**) HRTEM images and skeletonized images (width = 15 nm except for Dresser images for which width is equal to 30 nm) of studied kerogen characterized by contrasted structural features. For the ^13^C NMR spectra, the black circles indicate spinning side bands. The dashed line (90 ppm) shows the limit between the “aromatic” and “aliphatic” zones.

H/C atomic ratios are known to decrease with the thermal maturation of kerogens (Lis *et al.*, 2006; Vandenbroucke and Largeau, 2007). In the present sampling set, H/C varies from 1.32 to 0.28 (Table 2). For the three samples dated at 0.42 Ga (n°3–5; Table 2), a classical decrease in H/C with metamorphism grade was observed. However, such a trend is not visible when comparing the lower greenschist samples (n°6–9) with the lower to mid-greenschist samples (n°10–14) or with the greenschist samples (n°15 and 16). In addition, the Middle Marker (n°16) and the Gunflint (n°8) samples exhibit anomalously high H/C values (1.32 and 0.75, respectively) especially when considering their NMR-derived aromaticity (98% and 92%, respectively; Table 2; Fig. 2a). This illustrates the fact that hydrogen-rich minerals may survive the acidic treatment. Indeed, ash contents often exceed 50% in kerogen residues (n°8, 9, 10, 12, 13, 15, and 16; Table 2). However, it must be noted that within a given metamorphic grade (such as the Farrel Quartzite samples that underwent greenschist facies metamorphism), no relationship exists between ash content and H/C ratio. In all samples except Middle Marker, the H/C ratio remains lower than 1 even though the ash content is quite high, which indicates that the samples underwent early catagenesis to metamorphism (Durand and Espitalié, 1976). Moreover, neither the H/C ratio nor NMR-derived aromaticity allows for distinction between non-Archean and Archean samples. Because of the presence of remnant iron oxide in kerogens, aromaticity could not be assessed from NMR in most Archean kerogens. The Dresser sample, on the other hand, presents a surprisingly low aromaticity (57%) for an Archean Sample. However, this result is consistent with the release of significant amounts of aliphatic moieties by pyrolysis (Derenne *et al.*, 2008). Such aliphaticity may reflect the input of poorly ordered CM through hydrothermal circulation as recently demonstrated in the Apex chert (Marshall *et al.*, 2012; Sforna *et al.*, 2014) or late pyrobitumen generation formed through carbonization (Bernard *et al.*, 2012).

**Table 2.** Results from Elemental Analysis, ^13^C NMR, Raman Spectroscopy, and HRTEM on the Studied Cherts

			*Elemental analyses*					*Raman spectroscopy*			
*N°*	*Chert reference*	*Age (Ga)*	*H content (%)*	*C content (%)*	*H/C atomic ratio*	*Ashes (%)*	^*13*^*C NMR Aromatic carbon (%)*	*FWHM-G*	*FWHM-D1*	*R1*	*HRTEM La (Å)*
1	Clarno	0.05	2.82 ± 0.2	44.8 ± 0.4	0.76	n.d.	66 ± 3	97 ± 3	249 ± 12	0.60 ± 0.02	4.9 ± 0.1
2	Rhynie	0.4	2.13 ± 0.2	31.3 ± 0.4	0.81	41	72 ± 3	85 ± 6	232 ± 10	0.67 ± 0.11	5.5 ± 0.1
3	Zalesie Nowe	0.42	4.47 ± 0.2	57.6 ± 0.4	0.93	n.d.	58 ± 3	79 ± 2	207 ± 3	0.84 ± 0.04	5.1 ± 0.2
4	Żdanow	0.42	2.64 ± 0.2	69.4 ± 0.4	0.46	n.d.	98 ± 1	67 ± 4	224 ± 6	0.86 ± 0.03	5.7 ± 0.2
5	Döbra	0.42	1.75 ± 0.2	74.5 ± 0.4	0.28	n.d.	99 ± 1	57 ± 2	162 ± 6	0.97 ± 0.05	6.6 ± 0.8
6	Gunflint	1.9	2.80 ± 0.2	66.0 ± 0.4	0.51	17	98 ± 3	66 ± 4	178 ± 6	0.77 ± 0.06	5.4 ± 0.6
7	Gunflint	1.9	1.2 ± 0.2	26.4 ± 0.4	0.55	41	n.d.	62 ± 3	180 ± 3	0.57 ± 0.01	n.d.
8	Gunflint	1.9	1.69 ± 0.2	27.2 ± 0.4	0.75	50	92	52 ± 2	175 ± 11	0.47 ± 0.01	6
9	Rietgat	2.65	0.77 ± 0.2	17.3 ± 0.4	0.53	62	n.d.	68 ± 4	82 ± 4	1.20 ± 0.03	n.d.
10	Farrel Quartzite	3.0	0.68 ± 0.2	19.4 ± 0.4	0.42	70	n.d.	60 ± 7	66 ± 3	1.39 ± 0.04	n.d.
11	Farrel Quartzite	3.0	2.76 ± 0.2	60.6 ± 0.4	0.55	13	n.d.	69 ± 3	81 ± 4	1.25 ± 0.04	n.d.
12	Farrel Quartzite	3.0	0.67 ± 0.2	21.1 ± 0.4	0.38	60	n.d.	65 ± 4	66 ± 6	1.58 ± 0.09	8 ± 2
13	Farrel Quartzite	3.0	0.84 ± 0.2	32.9 ± 0.4	0.31	58	n.d.	58 ± 2	61 ± 1	1.58 ± 0.04	n.d.
14	Farrel Quartzite	3.0	0.99 ± 0.2	40.2 ± 0.4	0.30	43	n.d.	65 ± 4	64 ± 4	1.45 ± 0.04	7.5 ± 0.5
15	Josefsdal	3.3	0.43 ± 0.1	13.2 ± 0.3	0.39	81	n.d.	56 ± 4	59 ± 3	2.17 ± 0.34	9.4 ± 0.8
16	Middle Marker	3.4	1.94 ± 0.2	17.6 ± 0.3	1.32	54	98 ± 1	60 ± 2	61 ± 2	1.70 ± 0.10	9 ± 0.4
17	Dresser	3.5	2.92 ± 0.2	52.2 ± 0.4	0.67	22	57 ± 3	57 ± 3	87 ± 5	1.35 ± 0.09	6.2 ± 0.3

n.d. = not determined.

**FIG. 3.**
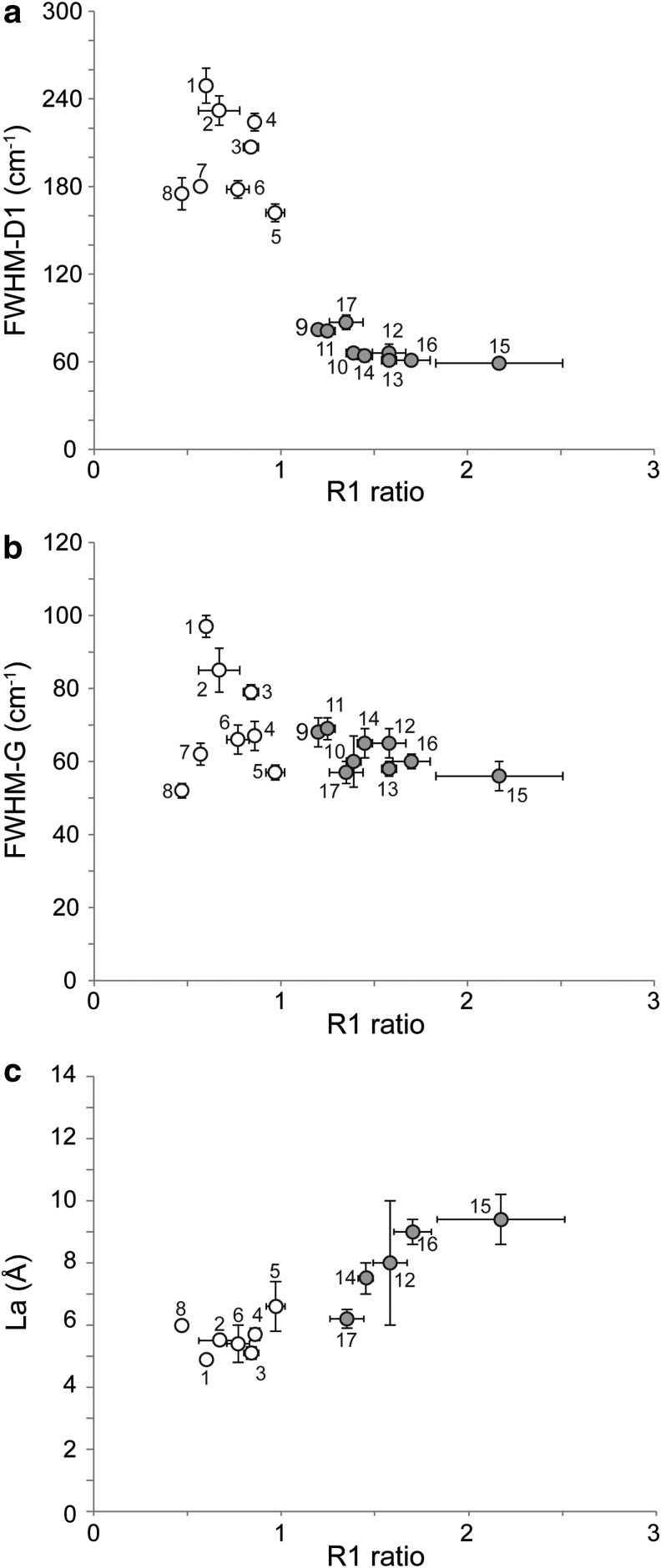
(**a**) Evolution of Raman R1 ratio vs. FWHM-D1, (**b**) evolution of Raman R1 ratio vs. FWHM-G, (**c**) evolution of Raman R1 ratio vs. HRTEM-derived mean La length. Non-Archean kerogens are represented by empty circles, whereas Archean ones are represented by gray circles.

Full width at half maximum of the D1 and G bands, together with the R1 ratios, was determined for each spectrum (Fig. 2b; Table 2). In this sample set, the R1 ratio clearly distinguishes non-Archean (0.47–0.97) from Archean samples (1.2–2.17; Fig. 3a). A similar distinction can be reached with FWHM-D1 (Archean FWHM-D1 = 59–87 cm^−1^ and non-Archean FWHM-D1 = 162–249 cm^−1^). A decrease in FWHM-G with increasing sample age and metamorphism was observed, although this parameter is less efficient in distinguishing between Archean and non-Archean cherts (Fig. 3b). FWHM-D1 and FWHM-G were previously reported to decrease with increasing natural carbonization (Wopenka and Pasteris, 1993; Bernard *et al.*, 2010; Rouzaud *et al.*, 2012). The gradual increase in the D1 band preponderance over the G band is consistent with the creation of defects inside the aromatic planes of the kerogen but without significant growth of the latter, which demonstrates that graphitization has not yet occurred. All samples fall into the range of FWHM-D1 (from *ca.* 300 to *ca.* 50 cm^−1^) and R1 ratio values (from *ca.* 0.5 to 2) corresponding to carbonization as previously defined for numerous natural and anthropogenic carbonaceous matters (Charon *et al.*, 2014; Romero-Sarmiento *et al.*, 2014). Nonetheless, FWHM-D1 and R1 ratios alone are not straightforward when distinguishing between samples that have undergone either the end of carbonization or the early beginning of graphitization when FWHM-D1 remains stable and R1 ratio decreases (Beyssac *et al.*, 2002). A HRTEM analysis is therefore required, as it provides an estimation of the mean length of polyaromatic layers and allows for distinction between carbonized and graphitized CM (Boulmier *et al.*, 1982; Charon *et al.*, 2014).

High-resolution transmission electron microscopy is a relevant tool with which to investigate the multiscale organization of CM (structure and nanostructure) by direct imaging of the profile of the polyaromatic layers (Boulmier *et al.*, 1982; Oberlin, 1984, 1989; Buseck and Huang, 1985; Bustin *et al.*, 1995; Rouzaud *et al.*, 2015, and references therein). The structure corresponds to organization at the atomic scale that results from the existence of polyaromatic layers, single or stacked, to form structural units; the fringe mean length then corresponds to the crystallite size La measured by Raman spectroscopy. The nanostructure is the organization from the nanometer to the micrometer scales. This results from the mutual orientation of layers or structural units to give domains of molecular orientation. The nanostructure provides information on the geochemical maturity, as shown for kerogens (Boulmier *et al.*, 1982; Romero-Sarmiento *et al.*, 2014; Rouzaud *et al.*, 2015). HRTEM images (Fig. 2c) reveal significant differences in multiscale organization within the studied series. Some samples such as Clarno are made of randomly oriented small (subnanometric) layers, indicating a low degree of thermal maturity. Others such as Middle Marker are made of much larger layers (some nanometers), which are stacked by 5 to 10 to form structural units. Moreover, the latter are locally oriented in parallel and form domains of molecular orientation. This means that these samples reached the metagenesis stage. Such characteristics are found for the oldest samples (Farrel Quartzite, Josefsdal, and Middle Marker) as well as for the most metamorphosed sample (Döbra), whereas the other samples appear much more disordered (see Clarno as an example in Fig. 2c). The mean length La derived from HRTEM image analysis ranges from *ca*. 5 Å up to 9 Å in the set of studied samples (Table 2; Fig. 3c). Thus, in agreement with Raman data, none of these samples underwent real graphitization (Tuinstra and Koenig, 1970; Ferrari and Robertson, 2000; Bernard *et al.*, 2010; Charon *et al.*, 2014), that is, a physical process corresponding to the triperiodic structure development and crystal growth that leads to the formation of perfectly stacked (along the A-B sequence) polyaromatic layers with mean length La up to 1000 nm (Oberlin, 1989). The increase in both La and R1 similarly reflects the increase in metagenesis and early metamorphism for the most mature (Fig. 3c). Temperature is a key controlling factor; time, pressure, and the source of the organic precursor are known to affect FWHM-D1 and the R1 ratio during carbonization (Lahfid *et al.*, 2010; Charon *et al.*, 2014). Positions of samples within the FWHM-D1 versus the R1 ratio diagram (Fig. 3a) clearly indicate that all Archean samples are still in the carbonization stage, which is in good agreement with their metamorphism facies. Hence, this implies that Archean kerogens recorded a more extensive carbonization than those that are non-Archean, which is in agreement with their higher metamorphic grade and age (Table 2) without having reached the graphitization stage.

## 4. Implications in the Search for Traces of Life

**FIG. 4.**
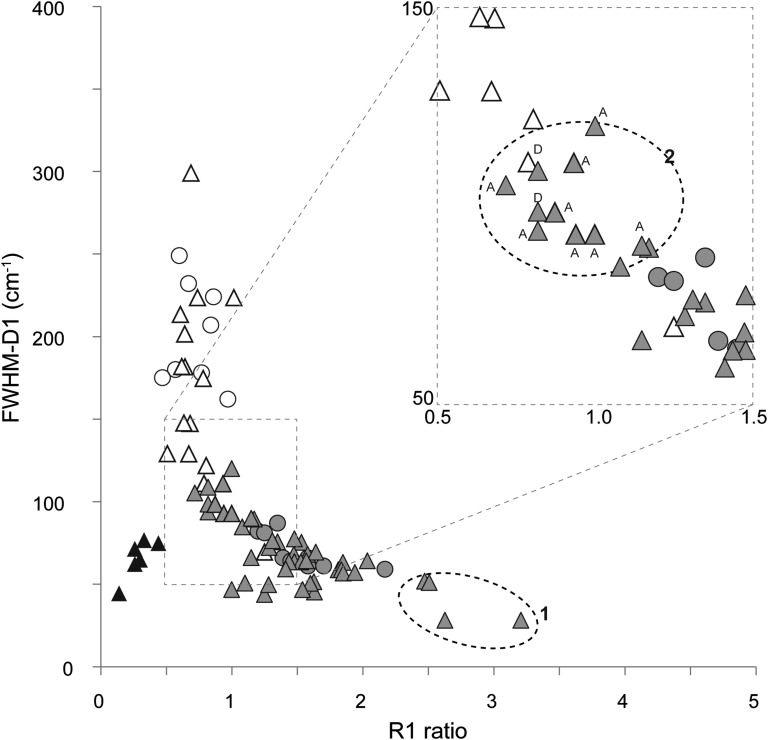
Compilation of R1 ratio and FWHM-D1 estimated on Archean and non-Archean CM from this study and from literature Raman spectra (Roberts *et al.*, 1995; Brasier *et al.*, 2002; Schopf *et al.*, 2002; Marshall *et al.*, 2005; Ueno *et al.*, 2006; Westall *et al.*, 2006; van Zuilen *et al.*, 2007; Igisu *et al.*, 2009; Javaux *et al.*, 2010; Papineau *et al.*, 2010; Sugitani *et al.*, 2010; Marshall *et al.*, 2012; Schiffbauer *et al.*, 2012; Lepot *et al.*, 2013; Noffke *et al.*, 2013; She *et al.*, 2013; Hofmann *et al.*, 2014). Non-Archean and Archean samples from this study are, respectively, represented by empty and gray circles, whereas those from the literature are respectively indicated by empty and gray triangles. Graphitized CM is represented by black triangles. Number 1 corresponds to samples with unusual high R1 (Ueno *et al.*, 2006; Hofmann *et al.*, 2014) discussed in the text. Number 2 indicates Archean CM with low structural order discussed in the text [D = CM from the Dresser Formation characterized in Noffke *et al.* (2013); A = CM from the Apex Basalt chert characterized in Brasier *et al.* (2002), Schopf *et al.* (2002), and Sforna *et al.* (2014)].

To test the general character of the aforementioned changes in CM structures as a consequence of carbonization, FWHM-D1 and R1 values taken from literature spectra on various Archean and non-Archean CM were added to the present set of results (Fig. 3). Data about graphitized kerogens from the 3.8 billion-year-old Isua Supracrustal Belt and Akilia rocks (van Zuilen *et al.*, 2007; Papineau *et al.*, 2010) were also added to highlight the impact of early “real” graphitization on FWHM-D1 and R1 ratio. These partially graphitized samples do not lie on the carbonization continuum reported in Fig. 4 and thus record strong structural changes, definitively altering the structure of original organic matter.

Literature Raman spectra of CM have been recorded by using a wide range of excitation laser energy (from 458 to 785 nm), and the relative intensities of the bands are known to strongly depend on the excitation laser energy (Mernagh *et al.*, 1984). However, no substantial differences in the determination of the R1 ratio were reported when comparing spectra obtained with a 514.5 nm laser (as in the present study) or with a 532 nm laser (Aoya *et al.*, 2010). As a result, literature Raman spectra acquired with a 514.5 or 532 nm laser were used in the following to obtain FWHM-D1 and R1 values and Fig. 4.

Taken together, the FWHM-D1 and R1 values confirmed the previously observed trend (Fig. 4). It must be noted that the same trend was observed on chondrites of petrologic type 3 (Bonal *et al.*, 2007; Busemann *et al.*, 2007), definitely showing that the carbonization continuum cannot be used to argue the biogenicity of CM (Pasteris and Wopenka, 2003). Regardless of the mineralogical context, two carbonization stages can be distinguished on the trend reported in Fig. 4. The first carbonization stage is characterized by a large range of FWHM-D1 values, which may reflect a precursor effect, the latter having a higher impact on carbonaceous organization (structure and nanostructure) at low temperature constraints (Lahfid *et al.* 2010). The beginning of the second stage of carbonization starts when R1 ratio reaches a value of *ca*. 0.8 to 1. Calibrating a Raman spectra–derived geothermometer on metasediments from the Glarus Alps (Cenozoic), Lahfid *et al.* (2010) pointed out that R1 ratio reached a value of 1 at about 300°C. Such a temperature seemed consistent with the metamorphism grade of studied rocks, which suggests that most Archean rocks were exposed to temperatures of roughly 300°C.

Full width at half maximum of the D1 band and R1 values also highlighted two groups of CM that merit further discussion (Fig. 4). A first group of CM falls at the outer limit of the carbonization/graphitization continuum, as the members of this group exhibit unusual and very high R1 ratio (≥2.5; Fig. 4). They comprise “coccoid-like” abiotic CM from the Cleaverville Formation (3.0 Ga; Ueno *et al.*, 2006) and some CM from the Kromberg and Hooggenoeg Formation cherts (3.48–3.26 Ga; Hofmann *et al.*, 2014). Although these high R1 values reflect a strong thermal alteration, this is not necessarily the case for all CM from South African Archean rocks that have undergone variable degrees of regional metamorphism (van Zuilen *et al.*, 2007). However, an intense hydrothermal activity was recorded for the samples as presented in Fig. 4 (Sugitani *et al.*, 1996; Hofmann *et al.*, 2014; Westall *et al.*, 2015). Although high R1 values are likely to reflect a strong carbonization, we question the impact of hydrothermal fluids on the structure of CM. Despite the fact that hydrothermal circulation can supply low ordered CM in comparison to syngenetic CM (Marshall *et al.*, 2012; Sforna *et al.*, 2014), the impact of hydrothermal fluids on syngenetic CM structure remains poorly constrained. Hence, to the best of our knowledge, it seems that CM with high R1 ratio was altered, which makes these samples unsuitable for classical biomarker analysis, although some CM with high R1 ratio may contain other biosignatures (morphological, geochemical and organic, *e.g.*, Westall *et al.*, 2011). As a result, CM with high R1 values must be avoided in the search for molecular evidence of life. A second group of samples comprise some Archean CM from the Apex Basalt chert (3.49 Ga; Brasier *et al.*, 2002; Schopf *et al.*, 2002; Sforna *et al.*, 2014) and microbially induced sedimentary structures (MISS) from the Dresser Formation (3.48 Ga; Noffke *et al.*, 2013). These sample types exhibit a high FWHM-D1 and low R1 values compared to other Archean CM (see Fig. 4 inset), and they are therefore located at the boundary between Archean and non-Archean samples despite their old age. Their relatively low R1 values (0.72 to 1) suggest that this Archean CM underwent milder carbonization. In turn, this should favor the conservation of molecular remnants of early traces of life.

## 5. Conclusion

Combining Raman spectroscopy, ^13^C NMR, HRTEM, and elemental analyses highlights the impact of carbonization in the structural order of non-Archean and Archean kerogens. The quantitative parameters FWHM-D1 and R1 ratio derived from literature Raman spectra indicate that most non-Archean CM underwent a first carbonization stage, whereas most Archean samples underwent a second carbonization stage. The latter is characterized by an increase in the R1 ratio, which probably reflects two stages of heteroatom release.

The existence of CM with unusually high R1 ratio values was highlighted. In some cases, hydrothermal activity is presumed to play a key role in maturation of CM by favoring the rise of defects in polyaromatic layers. In the carbonization continuum, some Archean samples fall at the boundary defined by CM of nonquestionable biological origin. Definitively non-graphitized, these samples are the best candidates to consider in the search for molecular biosignatures.

## Abbreviations Used

CM: carbonaceous matter
FWHM: full width at half maximum
HRTEM: high-resolution transmission electron microscopy
NMR: nuclear magnetic resonance
